# Post-translational changes in *Phialophora verrucosa via* lysine lactylation during prolonged presence in a patient with a *CARD9*-related immune disorder

**DOI:** 10.3389/fimmu.2022.966457

**Published:** 2022-08-08

**Authors:** Yinggai Song, Xiao Liu, J. Benjamin Stielow, Sybren de Hoog, Ruoyu Li

**Affiliations:** ^1^ Department of Dermatology and Venereology, Peking University First Hospital, Beijing, China; ^2^ Research Center for Medical Mycology, Peking University, Beijing, China; ^3^ National Clinical Research Center for Skin and Immune Diseases, Beijing, China; ^4^ Radboud UMC: Radboud University Medical Center/CWZ Center of Expertise for Mycology, Nijmegen, Netherlands; ^5^ Foundation Atlas of Clinical of Fungi, Hilversum, Netherlands

**Keywords:** Post-translational modification, lysine lactylation, CARD9, micro-evolution, melanin

## Abstract

*CARD9*-related inherited immune disorders are a major risk factor for chronic disseminated fungal infection. In addition to pathogens of *Candida* and dermatophytes, the environmental opportunists of the black yeast-like fungi are relatively frequent in this patient cohort. Particularly the genus *Phialophora* is overrepresented. We investigated two isolates of a strain of *P. verrucosa* residing in a *CARD9* patient, sampled with a period of ten years apart. Genomes, melanization and antifungal susceptibility of progenitor and derived strains were compared, and potential adaptation to the host habitat was investigated with proteomic techniques using post-translational modification as a proxy. Global lactylation analysis was performed using high accuracy nano-LC-MS/MS in combination with enrichment of lactylated peptides from digested cell lysates, and subsequent peptide identification. The genome of the derived isolate had accumulated 6945 SNPs, of which 31 were detected in CDS. A large number of identified proteins were significantly enriched, e.g. in melanin biosynthesis. A total of 636 lactylation sites on 420 lactylated proteins were identified, which contained in 26 types of modification motifs. Lysine lactylation (Kla) was found in 23 constituent proteins of the ribosome, indicating an impact of Kla in protein synthesis. Twelve lactylated proteins participated in pathogenicity. A protein-protein interaction (PPI) network analysis suggested that protein lactylations are widely distributed influencing various biological processes. Our findings reveal widespread roles for lysine lactylation in regulating metabolism and melanin biosynthesis in black fungi. Several large rearrangements and inversions were observed in the genome, but genomic changes could not be linked to adaptation or to known clinically relevant properties of progenitor to derived isolate; *in vitro* antifungal susceptibility had largely remained unaltered.

## Introduction

The discovery of rare inherited immune disorders, such as the one accompanying mutations in the caspase recruitment domain-containing protein 9 gene (*CARD9*), led to a paradigm change for the understanding of a number of severe fungal infections. The CARD9 protein takes an important role in the innate immune system in multifunctional signaling complexes for the recognition of virus, bacteria, parasites and fungi (1, 2) and Song et al. (3) noticed that the spectrum of fungal species associated with dysfunctional CARD9 protein deviated significantly from the list of agents involved in other diseases, e.g. by the near absence of *Aspergillus*. In contrast, melanized fungi, which otherwise are considered to be coincidental opportunists, were overrepresented. This indicates a specialized response to the window of opportunity in the innate immunity provided by impaired *CARD9* signaling, which appears to be shared by *Candida*, dermatophytes and black fungi, but seems absent from *Aspergillus* (3).

One of the fungi investigated by Song et al. (3) was *Phialophora verrucosa*, a rare environmental fungus related to the black yeasts (order *Chaetothyriales*). This fungus has been reported from seven *CARD9*-related cases in China (4, 5). From one of the patients with a chronic infection for more than a decade, the fungus was isolated repeatedly. Identification of strains isolated with an intermittent period of ten years revealed that the same species was concerned. Given the rarity of *P. verrucosa* (6), and its fluctuating clinical course with re-emergence of the fungus upon acquiring resistance to successive antifungals, re-infection of different isolates seemed unlikely. This prompted a proteogenomic study to monitor eventual adaptations of a single strain during its prolonged presence in live human tissue.

Numerous studies have stressed that important biological functions may be influenced significantly by post-translational modifications. In the present paper we investigate whether differences between the two clinical isolates from different time points indicate (epi)genetic adaptation *via* posttranslational modifications enhancing survival in host tissue. Epigenesis, involving histone modifications that directly affect chromatin structure, have poorly been explored with respect to fungal infections. Several epigenetic mechanisms are known, such as DNA methylation (7), chromatin remodeling (8) by histone or polycomb/trithorax proteins (9) or modulation of gene expression by microRNAs (10). In addition, protein acetylation and methylation are dynamic posttranslational modifications (PTM) notable in hundreds of proteins (11). In addition to histones, proteins involved in numerous biological processes can be modified, influencing metabolism, translation, gene expression regulation, and oxidative stress response (12, 13).

Lysine lactylation (Kla), i.e. protein modification at lysine residues was reported as a new type of PTM (14), being involved in active gene expression in macrophages. Gao et al. (15) reported on a proteomic survey of lactylation in *Botrytis cinerea*, a destructive necrotrophic plant pathogen. Kla may lead to PTM *via* histone modulation, and may influence biological processes due to functional changes of metabolically active proteins. The present paper investigates the posttranslational effectors provoked by Kla in the opportunistic fungus *Phialophora verrucosa* during its presence *in vivo* over a period of ten years. In order to understand clonality between strains over time, genome sequences of both strains were compared and relative polymorphisms between isolates quantified. Subsequently, we applied high-performance liquid chromatography, Kla modified peptide enrichment and Orbitrap mass spectrometry to investigate for the first time adaptive post translational signatures in clinical fungi.

## Materials and methods

### Strains and culture conditions

Two *Phialophora verrucosa* strains (BMU 00512, BMU 04928) were isolated from facial and back skin tissues of a *CARD9*-deficient phaeohyphomycosis patient ten years apart and were preserved at –80°C in the Research Center for Medical Mycology, Peking University. For protein extraction, conidia obtained from agar slant cultures were grown (10^7^ conidia/mL) in 100 mL Sabouraud’s Glucose Broth (SGB) on a rotary shaker (200 rpm) at 28°C. Mycelia were harvested after 48 h, frozen in liquid nitrogen, and stored at −80°C until further processing. Three replicates were prepared and analyzed for each of the samples.

### Lactic acid and melanin

For the analysis of lactic acid production, 10^7^ conidia of *P. verrucosa* were inoculated into liquid SGB medium, and cultured at 28°C for 48 h in a rotary shaker (180 rpm) in darkness. Cultures were harvested, lysed and lactic acid in the samples was determined using ELISA (BC2235, Solarbio, Beijing) at wavelength 570 nm. Melanin was detected by using melanin quantitative detection kit (GMS50365.3, Genmed) at wavelength 490 nm following the manufacturer’s instructions.

### Antifungal susceptibility testing

AFST was performed by broth microdilution method according to CLSI M38-A3 protocol (CLSI, 2017). Amphotericin B (0.03-16 μg/ml) and fluconazole (0.25-256 μg/ml) were purchased from Sigma-Aldrich (St. Louis, USA), itraconazole (0.03-16 μg/ml) and voriconazole (0.03-16 μg/ml) from ShouguangFukang Pharmaceutical (Shandong, China), terbinafine (0.002-2μg/ml) from Qilu Pharmaceutical (Shangdong, China), and posaconazole (0.008-4 μg/ml), caspofungin (0.25-256 μg/ml) and micafungin (0.25-256 μg/ml) from HuazhongHaiwei GeneTechnology (Beijing, China). Cultures were grown on Potato Dextrose Agar (PDA), cells recovered in sterile saline containing 0.05% Tween-20 and diluted to 2–5 × 10^4^ CFU/ml with RPMI-1640. Growth in the wells was checked daily. MICs were determined by visual inspection compared with the growth controls on two separate occasions. Agreement meant discrepancies of no more than two dilutions. Abbreviations of compounds are according to de Hoog et al. (6).

### Genome analysis

#### DNA extraction and preparation

Mycelia were harvested from fresh cultures grown on Sabouraud’s Glucose Agar (SGA), frozen in liquid nitrogen and stored at −80°C until used. Samples were ground with liquid nitrogen and extracted with SDS (DNA was verified on agarose gel electrophoresis and quantified by Qubit^®^ 2.0 Fluorometer (Thermo Scientific). DNA was fragmented by sonication to a size of 350 bp, then DNA fragments were end-polished, A-tailed, and ligated with the full-length adaptor for Illumina sequencing with further PCR amplification. PCR products were purified (AMPure XP system) and libraries were analysed for size distribution by Agilent2100 Bioanalyzer and quantified using real-time PCR.

#### Genome sequencing and assembly

Genomic DNA extracts of BMU 00512 and BMU 04928 were sequenced with 1 μg DNA per sample as input material with an Illumina NovaSeq PE150 at the Beijing Novogene Bioinformatics Technology Co., using paired-end and mate-paired libraries. Sequencing libraries were generated using NEBNext^®^ Ultra™ DNA Library Prep Kit for Illumina (NEB, U.S.A.) following manufacturer’s recommendations and index codes were added to attribute sequences to each sample. Quality control of the reads was performed using FastQC v0.11.81 and PCR adapter reads and low-quality sequences were removed by BBMap2. High-quality reads were *de novo* assembled using SOAP (16, 17), SPAdes v3.10.0 (18) and ABySS (19) with default parameters for Illumina paired-end reads. The completeness of the genome assemblies was accessed by quantifying the presence of the Core Eukaryotic Genes (CEGs) *via* CEGMA (20).

#### Structural annotation

For gene prediction, the software Augustus (21) was used for generating training gene sets. The complete annotation pipeline, PASA, as implemented at the Broad Institute, was applied which involved the following steps: (1) *ab initio* gene finding using GeneMarkHMM, FGENESH, Augustus, SNAP, and GlimmerHMM. (2) protein homology detection and intron resolution was done with GeneWise software and the uniref90 non-redundant protein database. (3) known ESTs, full-length cDNAs, and Trinity RNA-Seq assemblies were aligned to the genome. (4)

EVidenceModeler (EVM) was used to compute weighted consensus gene structure annotations.

#### Functional annotation

Seven databases were used for gene prediction, i.e. GO (Gene Ontology), KEGG (Kyoto Encyclopedia of Genes and Genomes), KOG (Clusters of Orthologous Groups), NR (Non-Redundant Protein Database databases), TCDB (Transporter Classification Database), P450, and Swiss-Prot. A whole-genome Blast search (E-value < 1e-5, minimal alignment length percentage larger than 40%) was performed against above seven databases. The secretory proteins were predicted by the Signal P database and secondary metabolism gene clusters by antiSMASH. Genes for virulence and drug resistance analyses were analyzed with the PHI (Pathogen Host Interactions), DFVF (database of fungal virulence factors). Carbohydrate-Active enzymes were predicted by the Carbohydrate-Active enZYmes Database.

#### Genome comparison and SNP analysis

Analysis included genomic synteny, core and specific genes, gene families, SNPs (Single Nucleotide Polymorphisms), Indels (insertion and deletion) and SV (Structural Variation) annotation. Genomic alignment showing SNPs, Indels and SV was performed using MUMmer and LASTZ. Core and specific genes were analyzed by the CD-HIT rapid clustering software with a threshold of 50% pairwise identity and 0.7 length difference cut-off in amino acids. Blast was used to pairwise align all genes and eliminate redundancy by solar with gene family clustering using Hcluster_sg software.

The homologous genomes of BMU 00512 and BMU 04928 were compared for the degree of mutation over time. The genetic order in the scaffolds of BMU 00512 was used as standard.

### Proteome analysis

#### Protein extraction

Fungal material was grinded to powder using liquid nitrogen and transferred to a 5-mL centrifuge tube. Four volumes of lysis buffer (8 M urea, 1% Triton-100, 10 mM dithiothreitol [DTT], 1% Protease Inhibitor Cocktail (Roche cOmplete™) was added, followed by sonication three times on ice using a high intensity ultrasonic processor (Ningbo Scientz Biotechnol., Zhejiang, China). The remaining debris was removed by centrifugation at 20,000 *g* at 4°C for 10 min. Protein was precipitated with cold 20% trichloroacetic acid (TCA) for 2 h at –20°C. After centrifugation at 12,000 *g*, 4°C for 10 min, the supernatant was discarded. The remaining precipitate was washed three times with cold acetone. The protein was redissolved in 8 M urea and the concentration was determined with BCA kit (Tiangen, Beijing, China) according to the manufacturer’s instructions. For digestion, the protein solution was reduced with 5 mM DTT for 30 min at 56°C, alkylated with 11 mM iodoacetamide for 15 min at room temperature in darkness and then diluted to an urea concentration of less than 2 M. Finally, trypsin was added at 1:50 trypsin-to-protein mass ratio for the first digestion overnight and 1:100 trypsin-to-protein mass ratio for a second 4 h-digestion.

#### Tandem mass tag labeling

Tryptic peptides were dissolved in 0.5 M triethylammonium bicarbonate (TEAB). Each channel of peptide was labeled with its respective TMT reagent (ThermoFisher Scientific) according to manufacturer’s instructions and incubated for 2 h at room temperature. Five microliters of each sample were pooled, desalted and analyzed by MS to check labeling efficiency. Subsequently, samples were quenched by adding 5% hydroxylamine. The pooled samples were then desalted with a Strata X C18 SPE column (Phenomenex, Torrance, CA, U.S.A.) and dried by vacuum centrifugation.

#### Affinity enrichment

For pan-antibody-based Post-translational modification (PTM) enrichment, modified and tryptic peptides were dissolved in NETN buffer (100 mM NaCl, 1 mM EDTA, 50 mM Tris-HCl, 0.5% NP-40, pH 8.0) and incubated with pre-washed antibody beads (PTM-1404, PTM Bio) at 4°C overnight with gentle shaking. Then the beads were washed for four times with NETN buffer and twice with H_2_O. The bound peptides were eluted from the beads with 0.1% trifluoroacetic acid, and subsequently the eluted fractions were combined and vacuum-dried. For LC-MS/MS analysis, the resulting peptides were desalted with C18 ZipTips (Millipore) according to the manufacturer’s instructions (22).

#### Nano liquid chromatography and tandem mass spectrometry

The sample was fractionated into fractions by high pH reverse-phase HPLC using Agilent 300 Extend C18 column (5 μm particles, 4.6 mm ID, 250 mm length). Briefly, peptides were separated with a gradient of 8% to 32% acetonitrile in 10 mM ammonium bicarbonate pH 9 over 60 min into 60 fractions. Then, the peptides were combined into 9 fractions and dried by vacuum centrifuging. The tryptic peptides were dissolved in solvent A (0.1% formic acid, 2% acetonitrile/in water), directly loaded onto a home-made reversed-phase analytical column (25-cm length, 75 μm i.d.). Peptides were separated with a gradient from 5% to 25% solvent B (0.1% formic acid in 90% acetonitrile) over 60 min, 25% to 35% in 22 min and climbing to 80% in 4 min then holding at 80% for the last 4 min, all at a constant flowrate of 450 nL/min on an EASY-nLC 1200 UPLC system (ThermoFisher Scientific). Chromatographic columns were packed with 1.9 μm/120 Å ReproSil-PurC18 resins (Dr. Maisch GmbH, Ammerbuch, Germany). The separated peptides were analyzed in Q ExactiveTM HF-X (ThermoFisher Scientific) with a nano-electrospray ion source. The electrospray voltage applied was 2.0 kV. The full MS scan resolution was set to 120,000 for a scan range of 350–1600 m/z. Up to 20 most abundant precursors were then selected for further MS/MS analyses with 30 s dynamic exclusion. The HCD fragmentation was performed at a normalized collision energy (NCE) of 28%. The fragments were detected in the Orbitrap at a resolution of 30,000. Fixed first mass was set as 100 m/z. Automatic gain control (AGC) target was set at 1E5, with an intensity threshold of 3.3E4 and a maximum injection time of 60 ms.

#### Protein identification

The resulting MS/MS data were processed using the MaxQuant search engine (v1.6.15.0) Tandem mass spectra were searched against the database obtained from the predicted proteins derived from the full genome sequence of the investigated strains (Phialophora_verrucosa_39412_TX_BMU5_combine_20201207_cdhit1.fasta), comprising a total of 27369 protein sequences. Redundant sequences from all available *P. verrucosa* genomes were filtered *via* cd-hit at a similarity threshold of 1 and an anti-library created which served to define the false discovery (FDR) by random matching, and a common contamination library was added to the database to eliminate the influence of contaminating proteins in the identification results.

#### Gene ontology

Proteins were classified by GO annotation using eggnog-mapper software (v2.0) into three categories: biological process, cellular compartment and molecular function. The software is based on the EggNOG database; the majority of comparisons applied *Cladophialophora carrionii* (TaxID 86049). For each category, a two-tailed Fisher’s exact test was employed to test the enrichment of the differentially modified protein against all identified proteins. GO with a corrected P-value < 0.05 was considered significant.

#### Pathway analysis

Kyoto Encyclopedia of Genes and Genomes (KEGG) database was used to identify enriched pathways by a two-tailed Fisher’s exact test to test the enrichment of the differentially modified proteins against all identified proteins. Pathways with a corrected P-value < 0.05 were considered significant. These pathways were classified into hierarchical categories according to the KEGG website.

#### Enrichment of protein domains

For each category proteins, the InterPro database providing functional protein analysis was researched and two-tailed Fisher’s exact tests were employed to test the enrichment of the differentially modified proteins against all identified proteins. Domain annotation was based on the Pfam database and the corresponding PfamScan tool. Protein domains with a corrected P-value < 0.05 were considered significant. MoMo (23) based on the motif-x algorithm was used to analyze motif characteristics of the modification sites. Peptide sequences consisting of 10 amino acids upstream and downstream of all identified K modification sites (6 upstream and downstream for phosphorylation modification) compared to all potential K modification sites in the species. When the number of peptides in a certain characteristic sequence form is greater than 20, and the statistical test P value is less than 0.000001, the characteristic sequence form is considered to be a motif of modified peptides. Frequency change scores [DS = −log_10_ (p value) × sign (diff. percent)] of amino acids near the modification site were presented in a heatmap.

#### Enrichment-based clustering

For further hierarchical clustering based on differentially modified protein functional classification (GO, Domain, Pathway, Complex), we first collated all categories obtained after enrichment along with P values, and then filtered for those categories which were at least enriched in one of the clusters with P value <0.05. This filtered P value matrix was transformed by the function x = −log10 (P value). Finally, these x values were z transformed for each functional category. The z scores were then clustered by one-way hierarchical clustering (Euclidean distance, average linkage clustering) in Genesis. Cluster memberships were visualized by a heat map using the “heatmap.2” function from “gplots” in the R-package. WolF Psort (24) was used for structural annotation.

#### Protein-protein interaction network

All differentially expressed modified protein database accessions or sequences were searched against the STRING database (v11.0) for protein-protein interactions, using *Cladophialophora carrionii* as model organism. There were 184 differential sites up-regulated corresponding to 106 proteins, and 267 down-regulated corresponding to 117 proteins; a total of 1148 interaction relationships were considered. Only interactions between the proteins belonging to the searched data set were selected, thereby excluding external candidates. STRING defines a metric called “confidence score” to define interaction confidence; we fetched all interactions that had a confidence score ≥ 0.7 (high confidence). An interaction network of the top 50 differentially modified proteins with the most closely interacting relationships according to degree of enrichment was visualized form STRING using “networkD3” in the R package using a confidence score > 0.7 (high confidence) of protein interactions. The PPI was visualized using cytoscape software v3.7.2.

#### Western blot assay

The mycelia of the tested strains were grown in SGB at 28°C for 48 h in a shaker. The proteins were extracted as mentioned above. Western blot was performed as previously described (25). Briefly, proteins with 20 μg protein/lane were separated by 12% SDS-PAGE and then transferred to polyvinylidene fluoride (PVDF) membranes. After blocking with 5% milk, immunoblotting was conducted using pan anti-Kla multiclonal antibodies at 1:1000 dilution (PTM-1401RM, Hangzhou, China).

## Results

**Figure 1:**
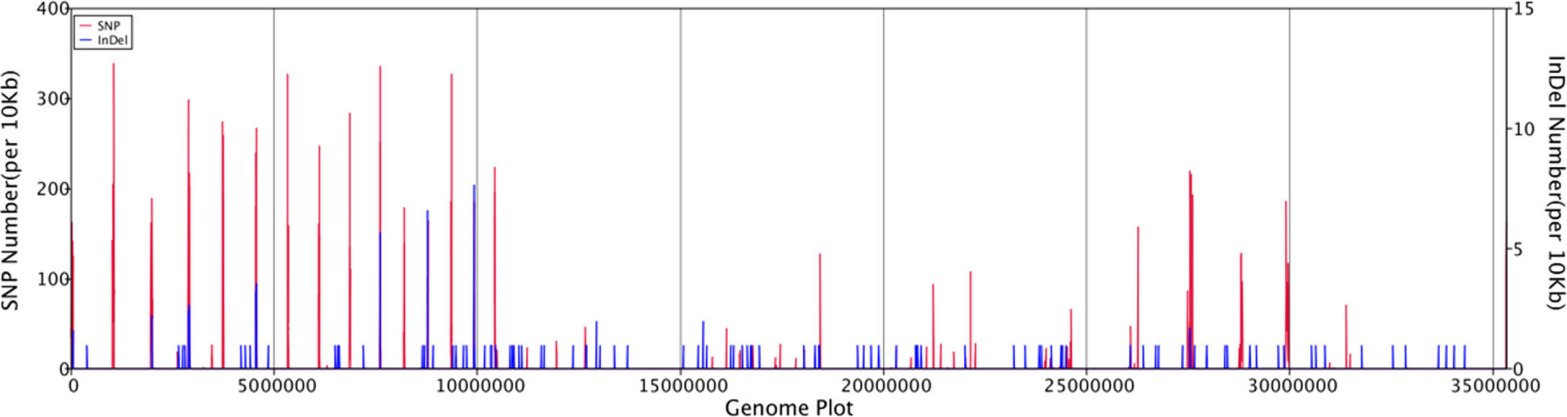
Distribution of SNPs and Indels along the genomes of isolates analyzed, with BMU 00512 taken as reference genome.

**Figure 2:**
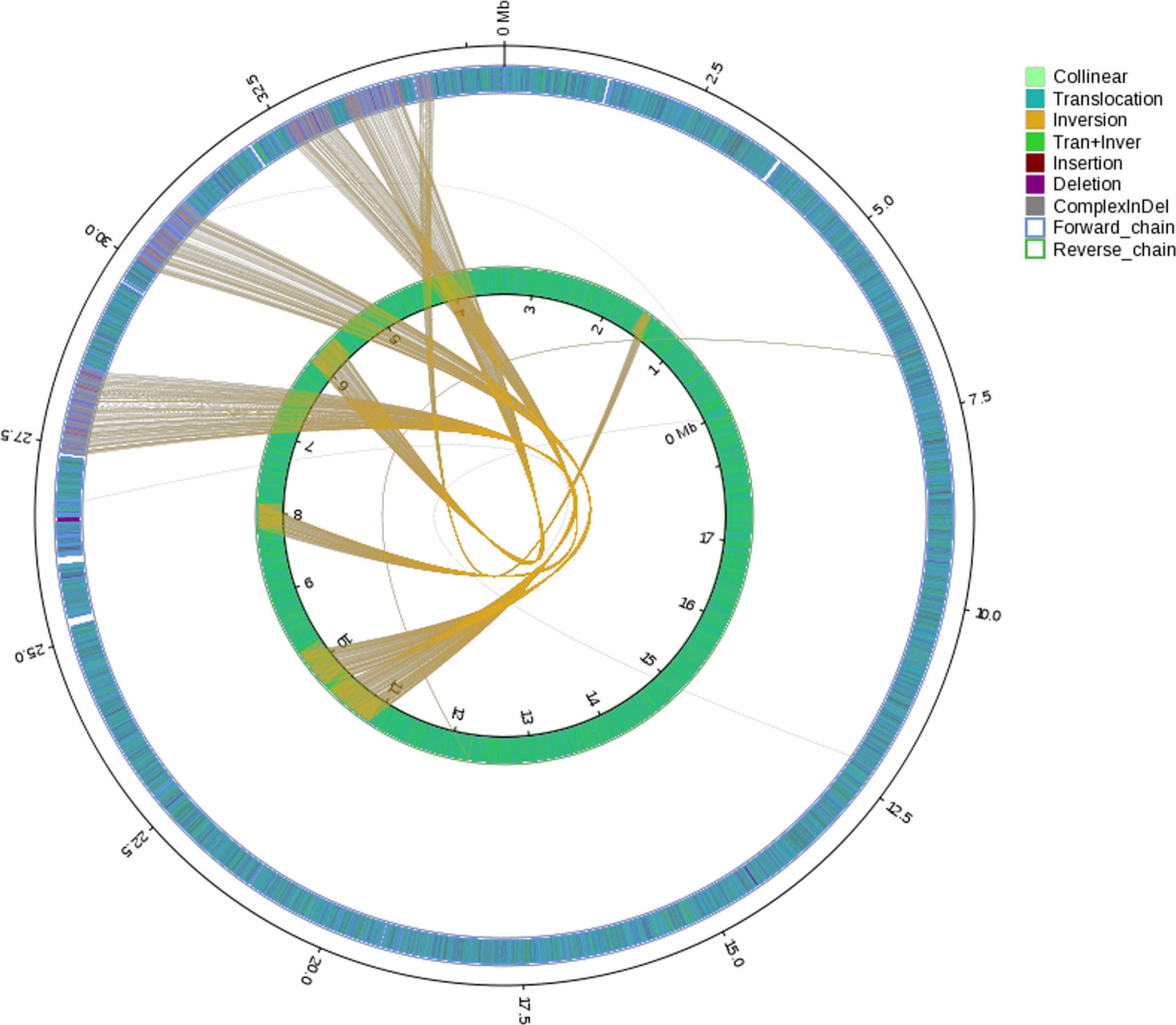
Rearrangements and inversions in genome BMU 04928 compared with BMU 00512 taken as reference genome. Collinear: synlinear region; Translocation: translocation region; Inversion: inversion region; Tran+Inver: translocation and inversion region; Insertion: insertion region with length ≥ 50 bp; Deletion: deletion region with length ≥ 50 bp; ComplexInDel: Regions that do not match but those locations correspond; Forward chain: the forward chain of the genome sequence, and the gene coordinates increase in the clockwise direction; Reverse chain: the reverse chain of the genome sequence, and the gene coordinates increase in the counterclockwise direction.

General genomic data are provided in **Table 1**. Genome sizes of strains BMU 00512 and 04928 were 34.0 and 34.2 mB, respectively. Percentages G+C varied from 53.57 to 53.58; similarity of the two strains was 99.95%. This value was slightly higher than when compared with an environmental strain of *Phialophora verrucosa*, BMU 07605, similarities with the strains under study being 99.18 and 99.69%, respectively. Comparison with BMU 07609 of *P. chinensis*, a member of the *P. verrucosa* complex, yielded similarity values below 93%. The genomes of BMU 00512 and 04928 were highly similar when sequence depth was compared (**Supplementary Figure 1**). **Figure 1** provides an overview of SNPs and Indels, which both show an uneven distribution along the genome. A total of 6945 SNPs was detected, of which 6782 were transitions and 163 transversions. The number of Indels was 111, of which 71 were insertions and 40 deletions. In the CDS, 31 SNPs were detected (**Supplementary Table 1**), of which 20 were non-synonymous, and 11 contained Indels (**Supplementary Table 2**). No mutations were detected in any of the known genes involved in antifungal resistance. This corresponded for AMB, CAS, FCZ, ITZ, MFG and PCZ with *in vitro* results of AFST, where strains BMU 00512 and 04928 deviated maximally with a single dilution step. With TBF and VCZ, three and two dilution steps difference were noted, respectively (**Supplementary Table 3**). Several large inversions were observed between BMU 00512 and BMU 04928 (**Figure 2**). However, it should be noted that large inversions can be a result of assembly ambiguities, which are to be resolved by larger library insert sizes; future confirmation with long-read sequencing is necessary.

**Table 1:** Genomic assembly and annotation statistics for BMU 00512 and BMU 04928.

BMU_04928	Scaffold	Contig
Total num (>500 bp)	58	126
Total length (bp)	34,207,930	34,202,081
N50 length (bp)	952,828	435,036
N90 length (bp)	271,107	159,600
Max length (bp)	3,541,721	2,028,093
Min length (bp)	55,978	2,600
Sequence GC%	53.58	53.58
**BMU_00512**	**Scaffold**	**Contig**
Total num (>500 bp)	55	74
Total length (bp)	34,034,191	34,034,001
N50 length (bp)	1,197,130	886.145
N90 length (bp)	243.659	208.497
Max length (bp)	2,431,464	2,287,921
Min length (bp)	50.078	2.971
Sequence GC%	53.57	53.57
All gene (#):	19636	
Pan gene (#):	10907	
Core gene(#):	7965	
Dispensable gene(#):	2942	
Strain specific genes:		
	Species ID	Number (#):
	BMU_00512	1418
	BMU_04928	1528
Similarity: 99.95%.		

The crude Coomassie-stained protein profiles of strains BMU 00512 and BMU 04928 were indistinguishable. Western blotting with antibodies against 2-hydroxyisobutyryllysine, crotonyllysine, succinyllysine and lactyllysine were all positive. In all blots, long exposure led to a stronger response in BMU 04928 than in BMU 00512 (**Supplementary Figure 2**).

Mass-to-charge ratios and signal intensities of peptides (primary spectrum) and ions after peptide fragmentation (secondary spectrum) were obtained by mass spectrometry analysis. A theoretical secondary spectrum database was constructed based on the protein sequences in the database, and the secondary spectrum generated by mass spectrometry was corrected after algorithm scoring and filtering. To increase the quality of the results, further data filtering was performed using a top-20 approach. The accuracy FDR (false discovery rate) of identification at three levels (spectrum, peptide, and protein) was set at 1%; at least one unique peptide must be included in the identification of each protein. The identified peptides and proteins after data filtering were as follows: total spectra generated by mass spectrometry were 57273, of which 9760 were effective spectra matching the theoretical secondary spectra; 6500 peptides were detected, of which 1922 (29.6%) were lactylated. A total of 760 proteins was identified. Of all proteins, 645 were up- and 587 were downregulated (ratio = 1.1). Of the lysine lactylated proteins, 106 proteins with 184 sites were upregulated, and 117 proteins with 267 sites were downregulated between BMU 00512 and BMU 04928 (ratio = 2.43; **Supplementary Figure 3**); the enriched modified proteins comprised 18.1% of the total.

**Figure 3:**
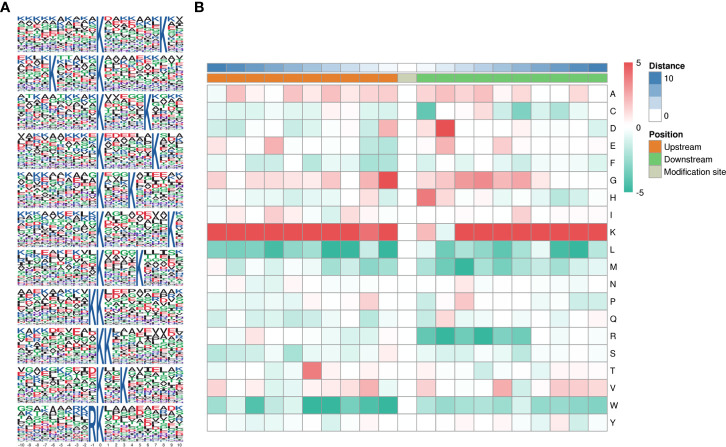
Pattern analysis of lactylated sites. **(A)** Peptide sequences of 10 amino acids upstream and downstream of lysine lactylated modification sites. **(B)** Comparison of all potential K modification sites in the species.

Motifs were analyzed using MoMo. Peptide sequences consisting of 10 amino acids upstream and downstream of all identified lysine lactylated modification sites were compared to all potential K modification sites in the species. When the number of peptides with a certain characteristic sequence was larger than 20 (P < 0.000001), the sequence was considered to be a motif of modified peptides. A total of twenty-six conserved amino acid sequences were extracted, of which the top 5 enriched ones were xxxxxxxxxx_K_xxxxxxxKxx, xxxxKxxxxx_K_xxxxxxxxxx, xxxxxxxxxx_K_xxxxxKxxxx, and xxxxxxxxxx_K_xxxxxxKxxx, and xxxxxxxxxx_K_xxxKxxxxxx (**Figure 3A**). To further analyze these motifs, heatmaps of the amino acid sequences around the lactylation sites were generated, which showed that certain amino acid residues surrounding the Kla were markedly enriched. K residues were observed to be enriched in the −10 to −1, and +3 to +10 positions; G residues were significantly enriched in positions −2 to −1, +2 to +6; H and D were significantly enriched in +1 and +2. Scores of frequency change (DS) of amino acids near the K modification site were calculated and displayed in a heatmap (**Figure 3B**).

Gene Ontology (GO) annotations were divided according to Biological Process, Cellular Component and Molecular Function. We performed differentially modified protein enrichment analysis for the three major categories in the GO classification. Differentially expressed modified proteins were compared with COG/KOG functional classification statistics. Significant enrichment of modified proteins was found particularly in cellular and metabolic processes, genes involved in cellular compartment, and binding and catalytic processes (**Supplementary Figure 4**). We subsequently divided the enriched lactylated proteins into 4 parts according to n-fold expression, called Q1 to Q4. For each Q group, GO classification, KEGG pathway and protein domain enrichment were analyzed, and cluster analysis was performed to find the correlation of protein functions with different differential expression folds in the comparison groups (**Supplementary Tables 4**–**6**).

**Figure 4:**
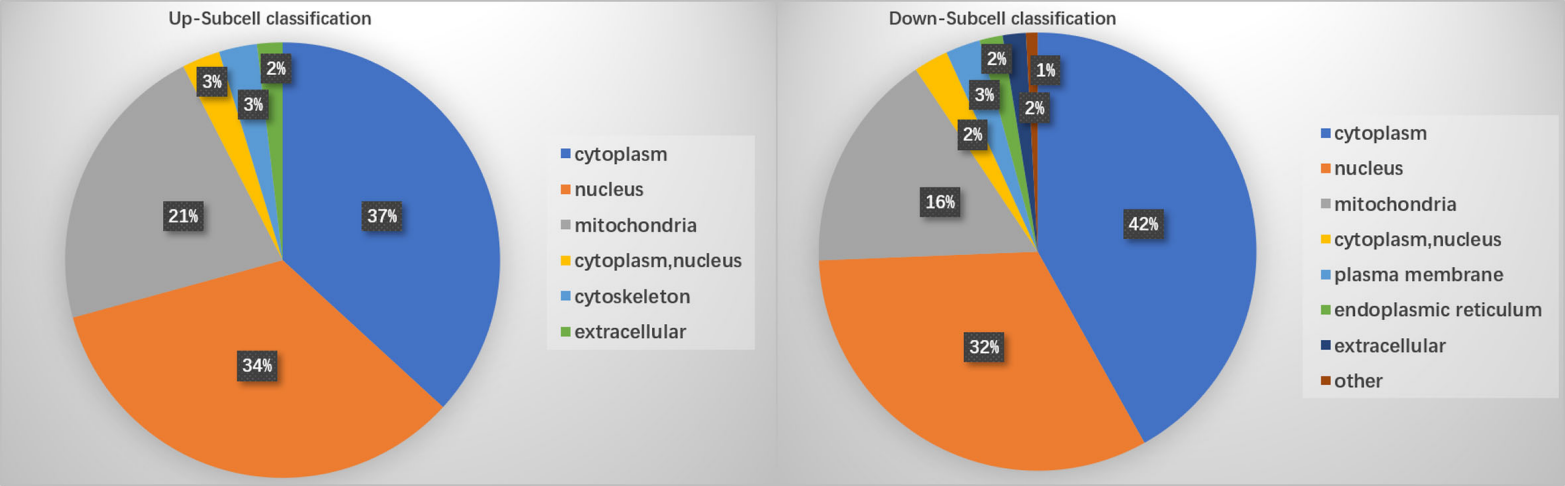
Enrichment of lactylated proteins distributed among subcellular localizations.

PTM lactylation analysis identified four sites in PKS by mass spectrometry. GO classification analysis annotated sites 211, 293, 234, and 325 in the PKS_ER domain-containing protein involved in catalytic activity. Further enrichment analysis on differentially expressed proteins involved in the KEGG pathway showed that PKS proteins were involved in tyrosine metabolism and were down-regulated in BMU 04928 compared to the wildtype strain BMU 00512. GO annotation also revealed that 123 histone sites were lactylated, and differential expression was classified in biological process. Only a single site was enriched between BMU 00512 and BMU 04928. For structural annotation of proteins in organelles, we used WolF Psort protein localization predictor. Enrichment analysis was carried out at the three levels of GO classification, KEGG pathways and protein domains, in order to reveal possible enrichment trends in functional types. Prevalence of proteins in subcellular locations were in line with those known for ascomycetous fungi (15) with the exception of cytoplasm with 39.3%. Up- *versus* downregulation of lactylated proteins according to subcellular compartments was nearly equal, with deviations slightly above 5% in cytoplasm (36.79–41.88%) and mitochondria (16.4–21.7%; **Figure 4**).

**Figure 5:**
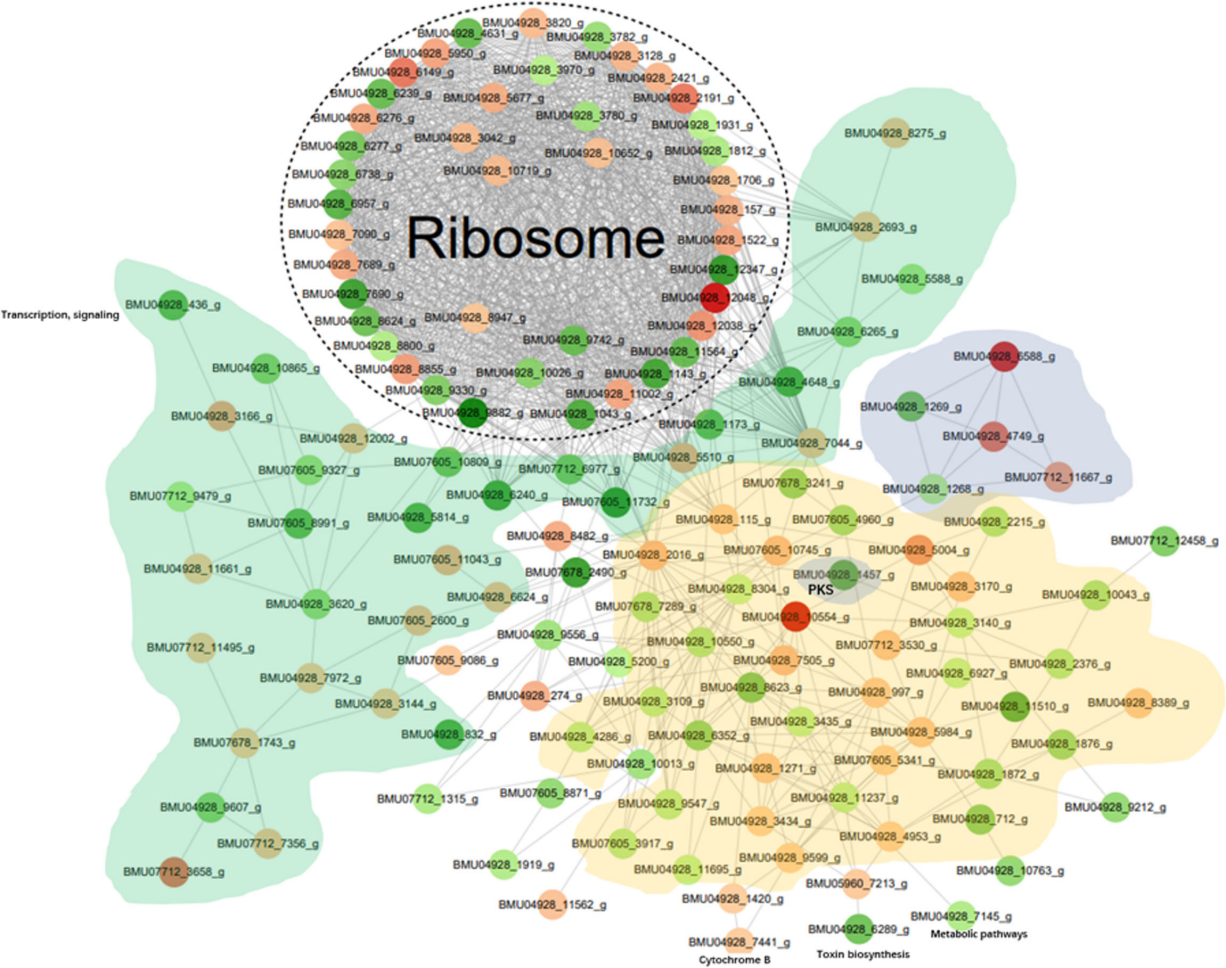
Protein-protein interaction (PPI) network of the 100 most up- and down-regulated lactylated proteins between BMU 00512 and BMU 04928. Ribosomal proteins form a dense cluster; proteins primarily involved in signaling, transcription and translation shown in green; metabolic proteins in yellow, histones in light grey, PKS in dark grey, heat shock proteins in pink.

The differentially modified proteins according to degree of enrichment were compared with the STRING (v11.0) protein interaction network database, using a confidence score > 0.7 (high confidence) of protein interactions. The data were visualized the “networkD3” tool in the R package. In order to clearly display the interaction between proteins, we selected the top-50 proteins with the most close edges (= links between nodes, i.e. the proteins) and inferred a protein-protein interaction network (PPI). The PPI network (**Figure 5**) shows a large number of closely interacting ribosomal proteins (white). The majority of selected nodes that are primarily involved in transcription, elongation and signaling (green) are downregulated. Nearly all nodes in a cloud involved with functions in metabolic pathways (yellow) are downregulated. Of the histones (grey), 2AB and 3 were downregulated, while H3 and particularly H4 showed significant upregulation. A cloud of heatshock proteins (pink) also responded variably, of which a J-domain containing protein was significantly downregulated. Also PKS-related proteins (dark grey) yielded variable responses; a PKS ER domain-containing protein involved in tyrosine metabolism was downregulated, while the conidial yellow pigment biosynthesis PKS involved in melanin synthesis showed an insignificant response. Comparison of *in vitro* melanin production between BMU 00512 and 04928 showed increased melanization in the latter (**Supplementary Figure 5**).

## Discussion

Inherited *CARD9-*related immune disorders and their clinical significance have first been reported somewhat over a decade ago (26, 27). Lanternier et al. (28) noted that black fungi were relatively prevalent upon *CARD9* impairment, observing that patients in a series of disseminated infections by the black yeast *Exophiala dermatitidis* all carried a homozygous mutation in the *CARD9* gene. CARD9 mediates signaling *via* PRR Dectin-1 and NF-κB leading to activation of pro-inflammatory (TNFα, IL-2, IL-6) and anti-inflammatory (IL-10) cytokines (29). Mutation of *CARD9* leads to impairment of the immune response against fungi. This previously unnoticed underlying disorder provided at least a partial explanation for some chronic and mutilating fungal infections in seemingly healthy individuals.

Numerous black fungi of the order *Chaetothyriales* are able to cause infections in humans (6), but frequencies are low. Their relatively high prevalence in patients with *CARD9*-related immune disorders (3) is as yet unexplained. Particularly members of the *Phialophora verrucosa* complex are overrepresented (3, 30). All *CARD9*-related infections have a chronic character and are poorly controlled by antifungal treatment (31), even if the respective antifungals are *in vitro* effective (32). In an attempt to understand this special preference of black yeasts, we monitored a patient with a chronic infection by *P. verrucosa* for more than ten years (5). To this purpose, a recent isolate (BMU 04928) was compared with an initial isolate obtained upon first presentation at the hospital (BMU 00512). The patient was initially treated with amphotericin B (cumulative dose 407.5 mg) and GM-CSF (300 × 10^4^ u/d) subcutaneously for 3 weeks. Because of AMB intolerance, therapy was changed to intravenous itraconazole injection 400 mg/d combined with oral terbinafine 500 mg/d orally. The skin lesions seemed to improve slightly, but later expanded and finally the patient expired.

The original wildtype strain, BMU 00512 was proven with barcoding genes (rDNA ITS and partial translation elongation factor 1-alpha; 3) to belong to the same species as BMU 04928 derived from the same patient ten years later. The rarity of this etiologic agent, and the identical Coomassie-stained whole-protein profiles of the two strains (**Supplementary Figure 2**) provided convincing evidence that a single strain was concerned, re-infection with another isolate of the same species being unlikely. The quantified amount of polymorphisms provides evidence for a number of mutations with an uneven distribution over the genome (**Figure 1**). While in clonal strains of ascomycetous yeasts, <100 to ~500 polymorphisms per year provides biological evidence for clonality (33), the amount of 6945 polymorphisms determined between our strains would be justifiable, assuming full mutational linearity. Of these mutations, only 31 were non-synonymous in CDS, affecting genes with various functions in biological process, molecular function and cellular component (**Supplementary Table 1**). The question arises whether in the absence of relevant mutations, other epigenetic effectors are impacting fungal survival, virulence and host adaptation. To study this, we investigated PTM as a proxy for potential proteomic changes. A plethora of mechanisms of histone modifications that affect chromatin structure are known, such as the polycomb/trithorax system, and modulation of gene expression by microRNA. The latter is impacted by methylation, phosphorylation, and acetylation (34). Lysine lactylation of histone proteins is a recently discovered mechanism of protein modification (14, 15). While it may appear counter-intuitive to assume strong linearity in genomic adaptation to the human host over a long period and with almost no detectable polymorphism, the biological significance of strong and detectable post-translational modifications such lactylation might enhance genomic stability, as is known e.g. in cancer biology from L- and D-lactate improving DNA and genomic stability of degenerated cells.

Using immunoblotting with antibodies to various lysine residues, we showed that PTM by hydroxyisobutyric acid (Khib), crotonic acid (Kcr), succinic acid (Ksucc), as well as lactic acid (Kla) occurs in the proteome, involving histones that interact with chromatins. Our data indicate, confirming those of Gao et al. (15), that Kla is widely distributed and also involves proteins other than histones, influencing various biological processes. In all Western blots, long exposure led to a stronger response in BMU 04928 than in BMU 00512 (**Supplementary Figure 2**), which suggests that the role of lysine modification had increased during ten years *in vivo*.

Ribosomal proteins were dominant in our samples (**Figure 5**) and this may have interfered with detection of proteins with low abundance which may have key biological functions. This is a methodical bias towards the results, potentially being influenced by the combination of liquid chromatography and mass spectrometry (e.g. competitive ionization). The interplay of the technical nodes requires additional investigation. We claim validity of our results because a large number of proteins was detected that are non-ribosomal, vary in molecular weight and (electro-)chemical properties, and accordingly perform analytically different in the designated LC-MS/MS assay when compared to the majority of ribosomal proteins. GO enrichment analysis demonstrated that the majority of the lactylated proteins were involved in cytoplasmic translation. Lactylated proteins were distributed in the cytoplasm, nucleus, and mitochondria (**Figure 4**), demonstrating occurrence of Kla in proteins with diversified cellular distribution. Kla dynamics are pronounced in pro-inflammatory M1 macrophages, which have an essential role in innate immunity against fungal infections (35). Further enrichment analyses of KEGG pathways yielded similar results, showing that the proteins associated with the ribosome were more likely to be lactylated, significantly impacting metabolic processes. GO analysis showed that histones were lactylated at 123 sites, but only a single site was significantly enriched between BMU 00512 and BMU 04928. Biochemical preference of enzymes for given substrates is largely determined by residues surrounding the modification site. The number of upregulated lactylated proteins between BMU 00512 and BMU 04928 was 106 with 184 sites, while 136 proteins with 207 sites were downregulated. Subcellular localization analysis of lactylated proteins did not significantly deviate between the two strains. Network analysis of the 100 most up- and down-regulated lactylated proteins showed that ribosomal proteins formed a dense cluster, with proteins involved in signaling, transcription and translation formed a cluster with longer edges (**Figure 5**). The histones, and the majority of the heatshock proteins formed a cluster, while the main cluster, with proteins involved in metabolic processes and secondary metabolite production formed a large assembly with rather long edges.

Of the lactylated proteins identified in this study, some have been reported to be involved in fungal virulence. Four sites were identified in PKS by mass spectrometry, which were annotated in GO analysis as located in the PKS_ER domain-containing protein involved in catalytic activity. Differential expression in KEGG showed that PKS proteins involved in the tyrosine pathway *via* DOPA to melanin polymerization were down-regulated in BMU 04928 compared with the original isolate BMU 00512. This was however not confirmed by *in vitro* analysis, showing an increase of melanization in BMU 04928. The downregulation is rather counter-intuitive, as melanin is generally judged to be a virulence factor protecting the cell against hyperoxygenic action of the macrophage (36, 37), and *in vitro* melanization had increased (**Supplementary Figure 5**). Song et al. (38) showed that subjecting cells of *Exophiala dermatitidis* may lead to loss of melanin, which corresponds with frequent occurrence of hyaline cells of melanized fungi in tissue (39). The halophilic black yeast *Hortaea werneckii* responded erratically to various stress factors (40). Possibly, melanin has a natural function in protection against irradiation (41), and may be less accountable as a general factor aiding virulence. Virulence and stress tolerance involve the activation of signaling cascades that result in a prolonged S-phase and delayed entry into mitosis. Recent studies indicate the involvement of unspecific PKSs and specific Cyclin Dependent Kinases (CDKs) in the eukaryotic stress response machinery. CDKs are core cell cycle regulators, and have been implicated in additional cellular processes. While our findings indicate involvement of lactylated enzymes, additional studies are required to reveal key nodes that may serve as lactylated master regulators of signaling cascades.

*Phialophora verrucosa* is probably an environmental opportunist (37). With prolonged presence in the stressful, non-optimal habitat provided by the human host, a certain degree of adaptive micro-evolution might be expected from progenitor (BMU 00512) to derived isolate (BMU 04928). During the decade of the fungus residing in our patient with *CARD9*-related immunodeficiency and fatal outcome, no evidence for genomic adaptation was recognizable, despite a significant number of SNPs, genomic rearrangements, and some non-synonymous mutations in CDS. However, lysine lactylation, used as a proxy for post translational mechanisms, was significantly enriched suggesting impact on a wide diversity of metabolic and regulatory processes.

## Data availability statement

The datasets presented in this study can be found in online repositories. The names of the repository/repositories and accession number(s) can be found below:

NCBI - PRJNA600036; ProteomeXchange PRIDE - PXD032921.

## Ethics statement

This study uses strains obtained from Peking University First Hospital. Peking University First Hospital ethics committee did not require the study to be reviewed or approved by an ethics committee because primarily isolated as part of my previous study for which ethical approval was obtained.

## Author contributions

RL and SH designed the experiments and supervised the data analysis. YS performed the experiments and wrote the relevant portions of the manuscript. XL and BS analyzed the data. All authors discussed the results and commented on the manuscript. All authors contributed to the article and approved the submitted version.

## Funding

The authors are grateful for the financial support of the National Natural Science Foundation of China (NSFC No. 81902043).

## Acknowledgments

We acknowledge Prof. Mihai Netea (Radboud University Medical Center) for advice concerning *CARD9* prevalence and Zhe Wan (Peking University First Hospital) in the identification of strains.

## Conflict of interest

The authors declare that the research was conducted in the absence of any commercial or financial relationships that could be construed as a potential conflict of interest.

## Publisher’s note

All claims expressed in this article are solely those of the authors and do not necessarily represent those of their affiliated organizations, or those of the publisher, the editors and the reviewers. Any product that may be evaluated in this article, or claim that may be made by its manufacturer, is not guaranteed or endorsed by the publisher.
