# Supplementary figures and images for: Post-translational changes in Phialophora verrucosa via lysine lactylation during prolonged presence in a patient with a CARD9-related immune disorder
Source: Front Immunol. 2022 Aug 8;13:966457. doi: 10.3389/fimmu.2022.966457 (PMC9395174; doi:10.3389/fimmu.2022.966457)

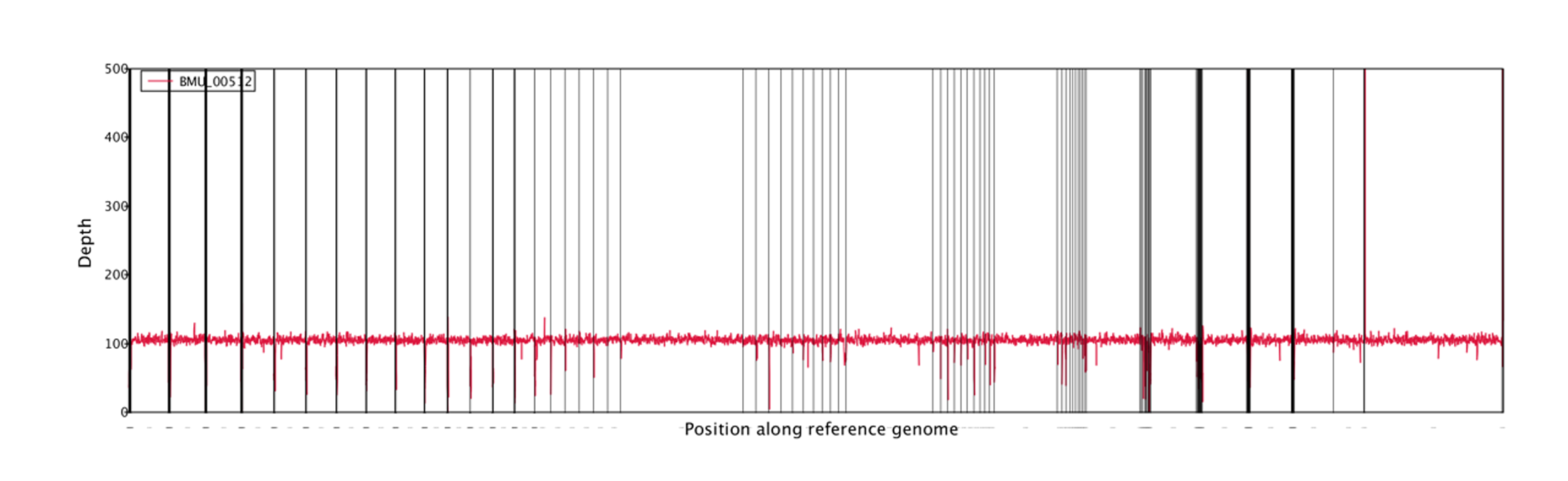

Supplement: Supplementary file 1 [file Image_1.tif]

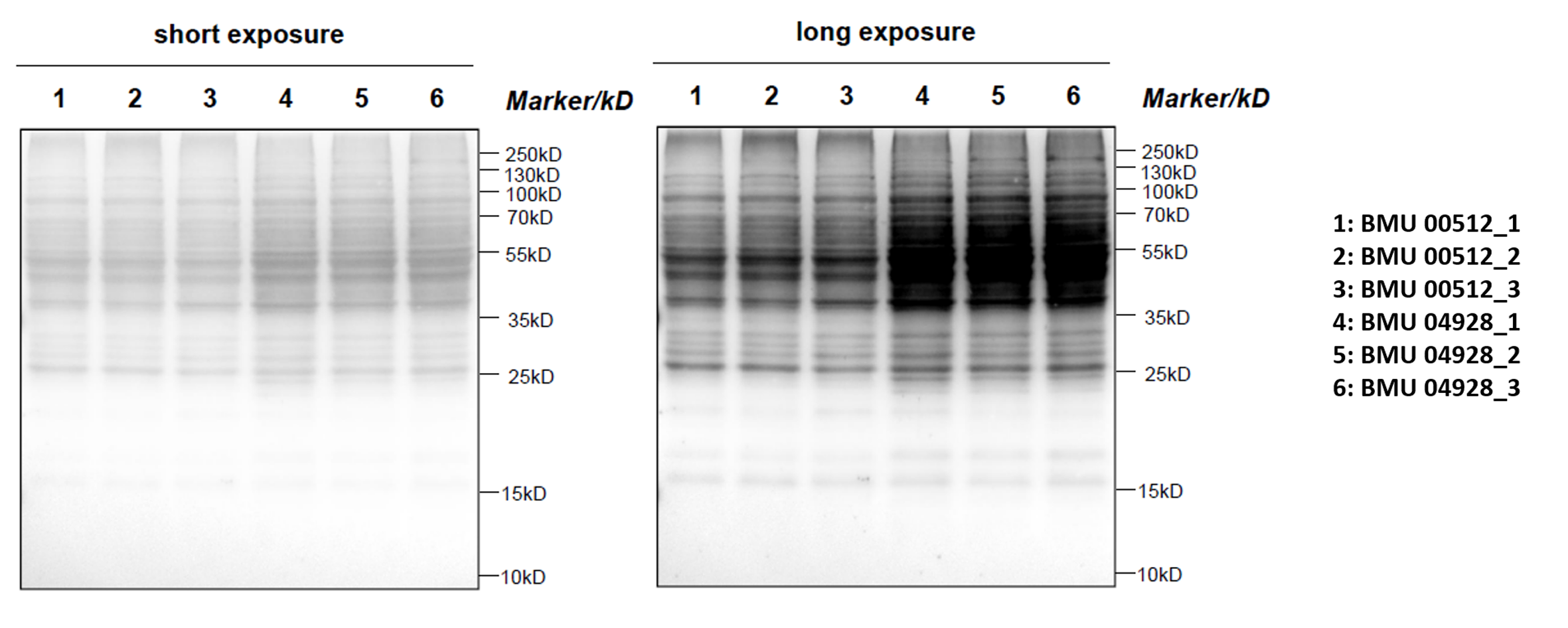

Supplement: Supplementary file 2 [file Image_2.tif]

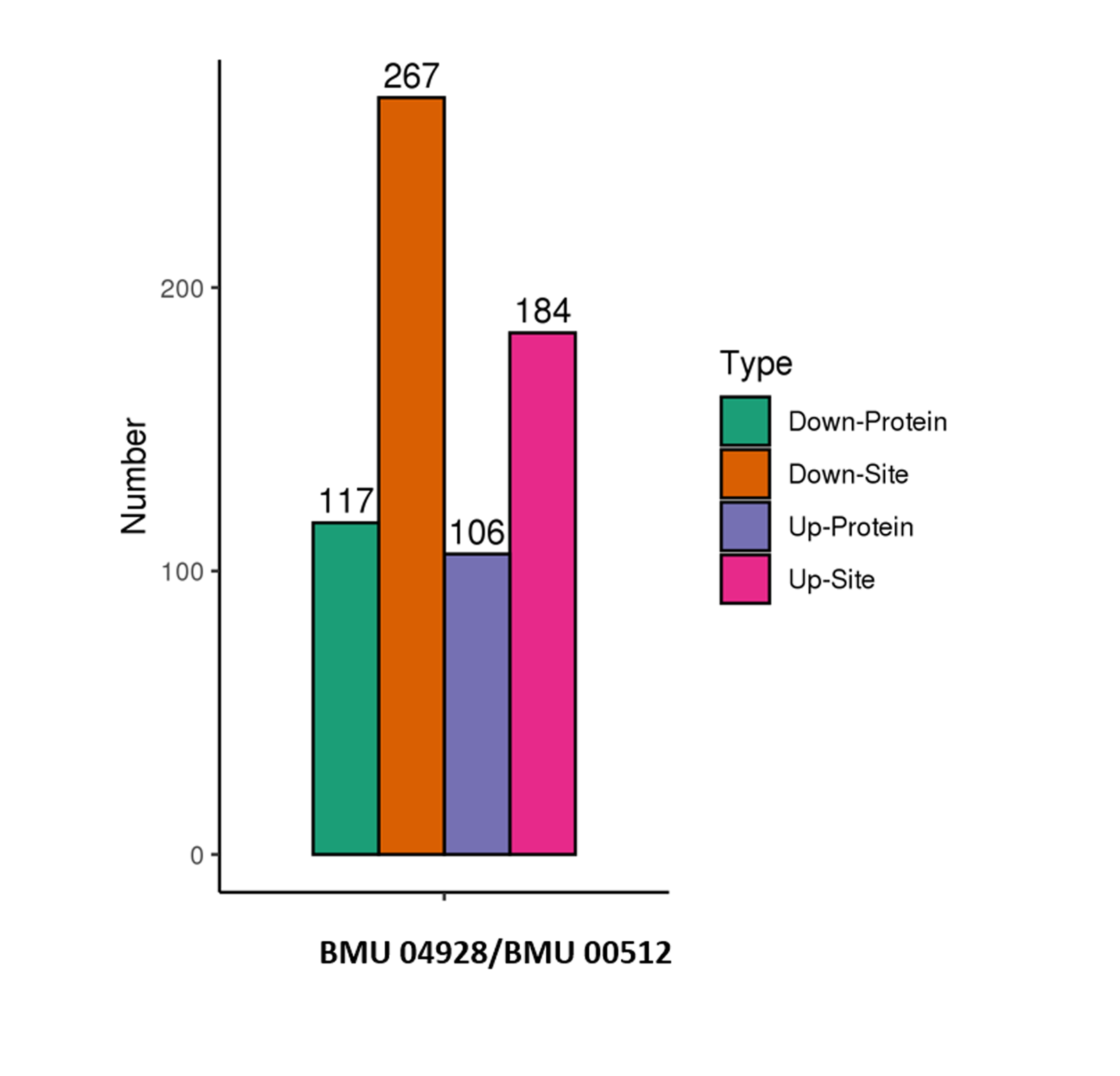

Supplement: Supplementary file 3 [file Image_3.tif]

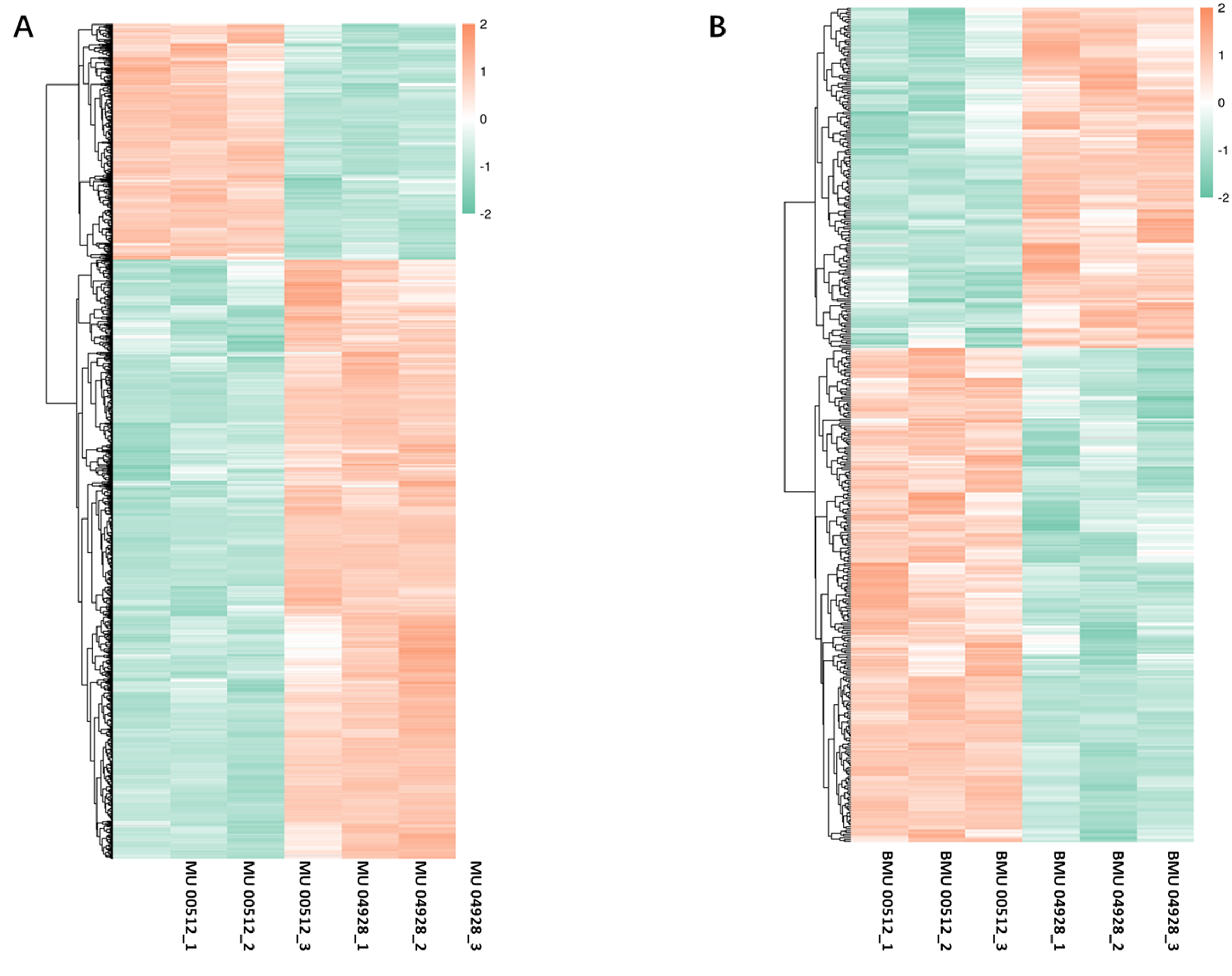

Supplement: Supplementary file 4 [file Image_4.tif]

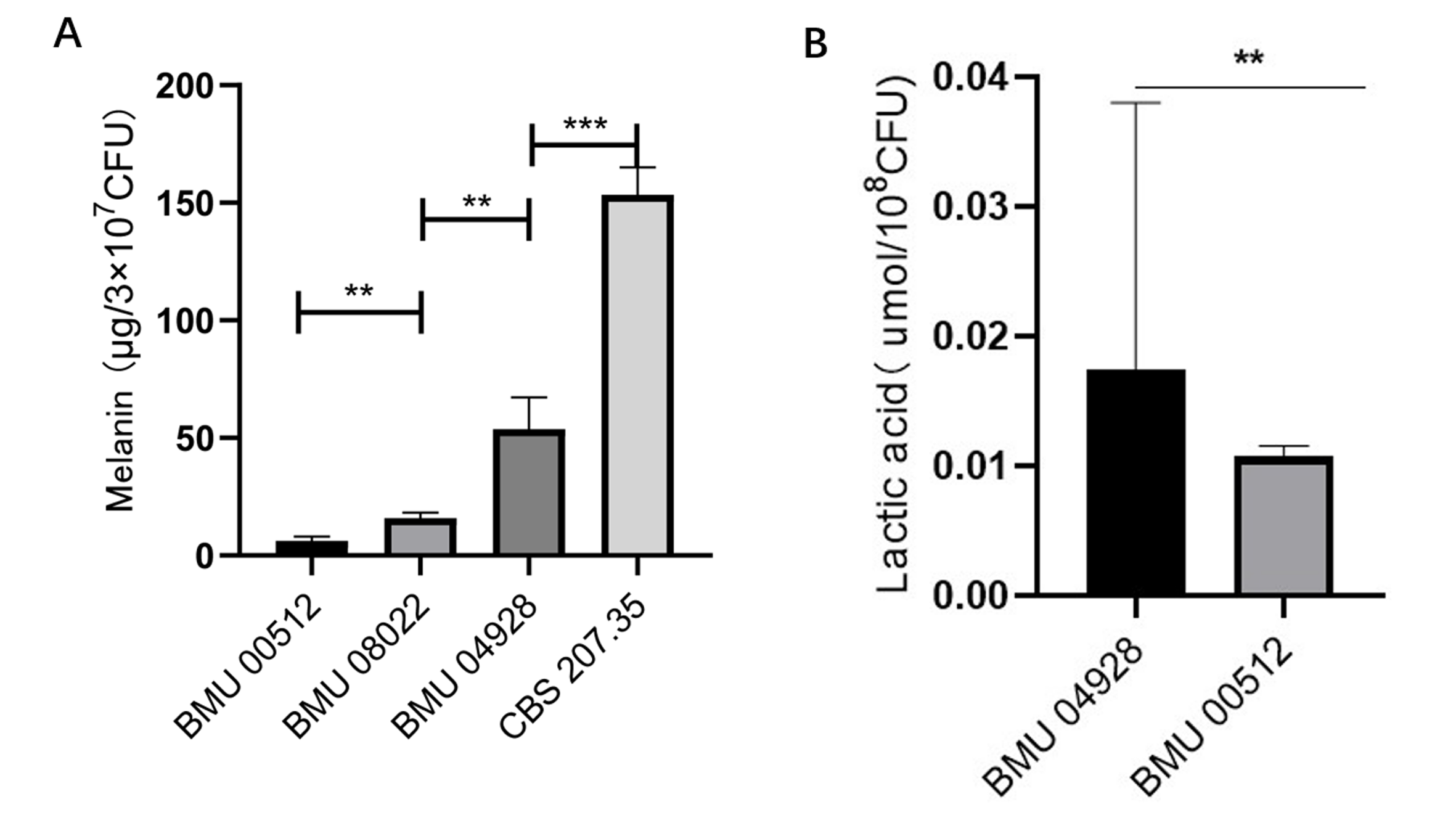

Supplement: Supplementary file 5 [file Image_5.tif]
